# Transcriptomic analysis reveals a critical role for activating G_s_α mutations in spontaneous feline hyperthyroidism

**DOI:** 10.1038/s41598-024-79564-z

**Published:** 2024-11-20

**Authors:** Thomas K. Hiron, Joana Aguiar, Jonathan M. Williams, Sara Falcone, Paul A. Norman, Jonathan Elliott, Robert C. Fowkes, Harriet M. Syme, Lucy J. Davison

**Affiliations:** 1 Department of Clinical Science and Services, The Royal Veterinary College, Hertfordshire, AL9 7TA UK; 2 Department of Physiology, Anatomy and Genetics, University of Oxford, Oxford, OX1 3PT UK; 3 Present Address: Dick White Referrals, Station Farm, London Road, Six Mile Bottom, Cambridgeshire, CB8 0UH UK; 4 Department of Pathobiology and Population Sciences, The Royal Veterinary College, Hertfordshire, AL9 7TA UK; 5 Department of Comparative Biomedical Science, The Royal Veterinary College, London, NW1 0TU UK; 6 Present Address: Department of Small Animal Clinical Sciences, Michigan State University College of Veterinary Medicine, East Lansing, MI 48824 USA

**Keywords:** Gene expression, Thyroid diseases

## Abstract

Feline hyperthyroidism (FHT) is a debilitating disease affecting > 10% of elderly cats. It is generally characterised by chronic elevation of thyroid hormone in the absence of circulating TSH. Understanding of the molecular pathogenesis of FHT is currently limited. However, FHT shares clinical and histopathological similarities with human toxic multinodular goitre, which has been associated with activating mutations in TSH receptor (TSHR) and G_s_α encoding genes. Using RNA-seq transcriptomic analysis of thyroid tissue from hyperthyroid and euthyroid cats, we identified differentially expressed genes and dysregulated pathways in FHT, many of which are downstream of TSHR. In addition, we detected missense variants in thyroid RNA-seq reads that alter the structure of both TSHR and G_s_α. All FHT-associated mutations were absent in germline sequence from paired blood samples. Only a small number of hyperthyroid cats demonstrated TSHR variation, however all thyroids from advanced cases of FHT carried at least one missense variant affecting G_s_α. The activating nature of the acquired G_s_α mutations was demonstrated by increased cAMP production in vitro. These data indicate that constitutive activation of signalling downstream of TSHR is central to the TSH-independent production of thyroid hormone in FHT, offering a novel therapeutic target pathway in this common disease.

**Supplementary Information:**

The online version contains supplementary material available at 10.1038/s41598-024-79564-z.

## Introduction

Hyperthyroidism is the most common feline endocrinopathy, and a major cause of morbidity in cats of middle age (7–8 years) and older^1–3^. It is typically caused by excessive production of thyroid hormone (T_3_/T_4_) from hyperfunctioning benign adenomatous thyroid nodules^1^. The prevalence of feline hyperthyroidism (FHT) has increased worldwide since its first description in 1979, however the reason for this increase remains unclear and is potentially multifactorial^2,3^. Genetic factors are likely to contribute given the decreased risk of certain breeds (such as Siamese and Burmese cats) developing FHT^4–6^. In addition, environmental factors such as diet, dietary contaminants (e.g., bisphenol A, polybrominated diphenyl ethers and flavonoids) and indoor living have been linked to development of hyperthyroidism in cats^5,7–10^. The major histopathological changes associated with disease are nodular hyperplasia of the thyroid, adenomatous hyperplasia and adenoma, while only 2% of hyperthyroid cats are diagnosed with thyroid carcinoma^11–13^. A range of therapies are available for FHT, including radioactive iodine therapy, thioureylene antithyroid drugs and thyroidectomy, however all available treatments carry risks, including the development of iatrogenic hypothyroidism. Median life expectancy for treated hyperthyroid cats is approximately 2–5 years from diagnosis^3,14^.

In normal thyroid tissue, circulating thyroid stimulating hormone (TSH) produced by the pituitary gland binds to its receptor (TSHR) on thyroid follicular epithelial cells, stimulating cell proliferation and thyroid hormone synthesis and release. TSHR is a G protein-coupled receptor (GPCR) which signals through the alpha subunit of G_s_ (G_s_α) to stimulate production of cAMP by adenylate cyclase. This results in activation of cAMP-dependent protein kinase A (PKA) and the transcription factor cAMP response element binding protein (CREB)^15,16^. Transcription of genes involved in thyroid hormone synthesis and proliferation is subject to a complex network of feedback and feedforward mechanisms^17^, and in addition to CREB, there are four transcription factors crucial to thyroid differentiation and function: Thyroid Transcription Factor 1 (TTF-1, encoded by *NKX2-1*), Thyroid Transcription Factor 2 (TTF-2, encoded by *FOXE1*), paired box gene 8 (encoded by *PAX8*) and hematopoietically expressed homeobox (encoded by *HHEX*). All of these proteins are directly or indirectly regulated by PKA^17^. TSHR can also couple with G_q/11_ to stimulate phospholipase C (PLC), leading to activation of protein kinase C (PKC). The precise role of G_q/11_-mediated TSHR signalling in the thyroid remains unclear. It has been shown to inhibit TSH-dependent thyroid hormone synthesis and thyrocyte proliferation in vitro^18^, but may be important for iodide organification and thyroid hormone secretion in response to TSH^19^. In hyperthyroid cats, circulating TSH concentrations are low due to homeostatic feedback inhibition, as a result of excess synthesis and secretion of thyroid hormones by thyroid follicular epithelial cells^20^. This results in toxic levels of thyroid hormone and systemic dysregulation of metabolism. The molecular events leading to autonomy of thyrocyte function in FHT have yet to be determined.

FHT shares histopathological and clinical similarities with toxic multinodular goitre (TMG), a common form of benign nodular thyroid disease affecting humans^13^. The major risk factors for TMG, besides advanced age and female sex, are iodine deficiency and mutations in the genes encoding TSHR or G_s_α^21^. These goitrogenic stimuli are believed to promote thyroid hyperplasia, eventually resulting in increased thyroid hormone synthesis independent of TSH. Iodine excretion is reduced in hyperthyroid cats compared to euthyroid cats, suggesting that iodine availability may also contribute to FHT, and both TSHR and G_S_α mutations have been identified in cats with FHT^22–24^. The most common cause of thyrotoxicosis in humans is Graves’ disease, an autoimmune disorder in which thyroid stimulating immunoglobulins (TSI) bind and activate TSHR, but several studies have shown that TSI are not present in FHT^25–27^. Given the clinical and histopathological similarities between species, detailed molecular characterisation of FHT may provide new insights into the pathogenesis of TMG in humans.

In this study, we used RNA-seq to identify differentially expressed genes in the transcriptome of thyroid nodules from clinically hyperthyroid cats compared to normal thyroid tissue from euthyroid cats. In addition, transcriptomic data was used to screen feline thyroid nodules for mutations in TSHR and G_S_α. To our knowledge, this is the first transcriptome-wide analysis of FHT, and even in humans these methods have only recently been applied to the study of TMG in a limited number of samples^28,29^. Identification of molecular mechanisms driving thyroid hyperplasia and autonomous production of thyroid hormone in FHT has the potential to reveal novel targets for treatment and prevention of this common disease.

## Results

### Identification of differentially expressed genes in FHT

**Fig. 1:**
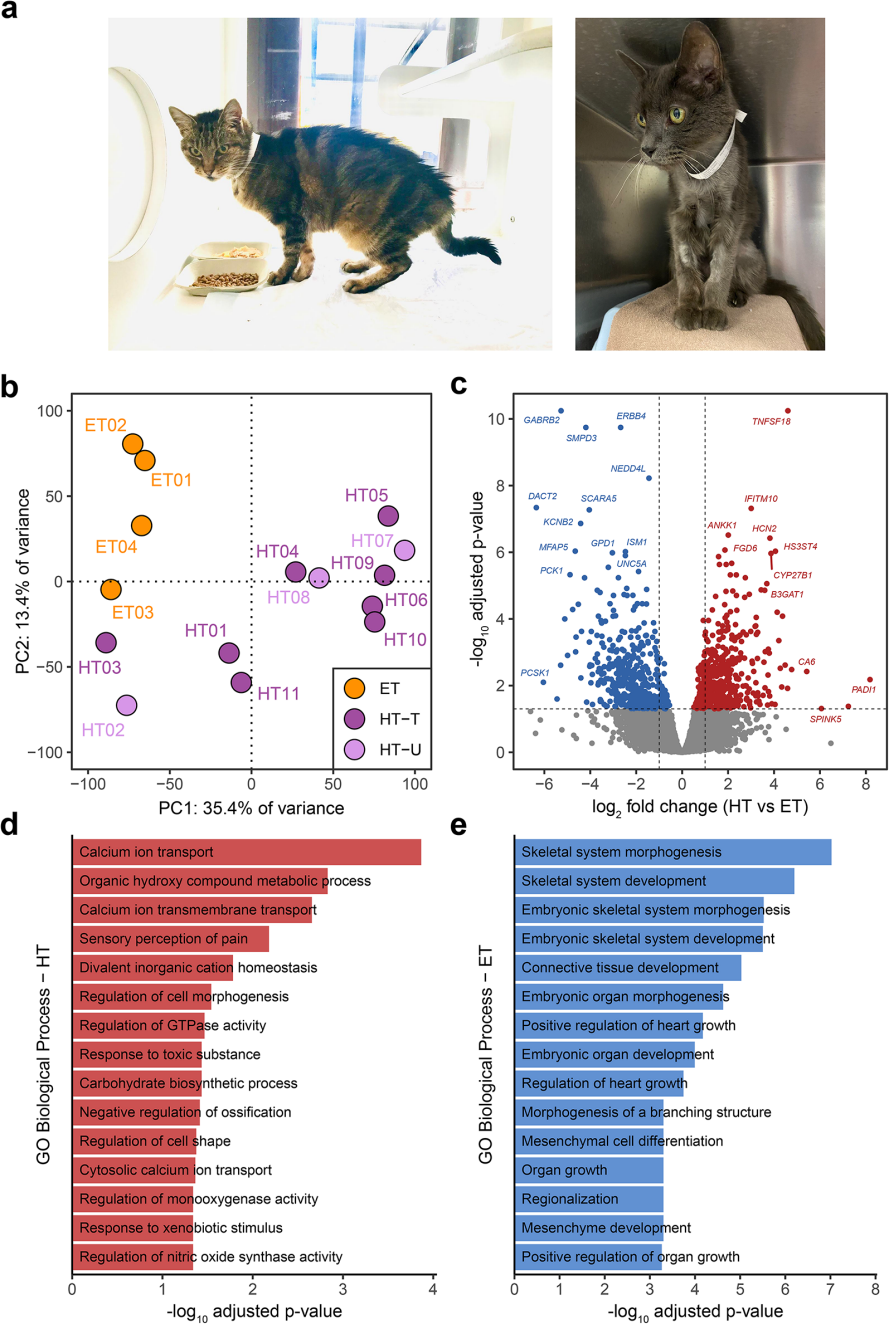
Identification of differentially expressed genes between all hyperthyroid (HT) and euthyroid (ET) samples. (**a**) Photographs of clinically hyperthyroid cats, with the unkempt coat and lean appearance observed in some FHT cases. (**b**) Principal component analysis of euthyroid (ET) and hyperthyroid RNA-seq samples from both treated (HT-T) and untreated (HT-U) cats. (**c**) Volcano plot showing differentially expressed genes (adjusted *p* < 0.05) with higher expression in ET (blue) or HT (red) samples. Grey points represent genes with no significant difference in expression between groups (adjusted *p* > 0.05). (**d**) and (**e**) Enriched Gene Ontology (GO) Biological Process terms for genes with higher expression in (**d**) HT samples and (**e**) ET samples.

Thyroid tissue samples were collected from cats with clinically diagnosed hyperthyroidism (Fig. 1a) and elderly euthyroid cats euthanised for reasons unrelated to this study. A summary of the clinical information for the cats included in the study is presented in Table S1. RNA was extracted from hyperthyroid (HT) and euthyroid (ET) samples and RNA-seq was performed. Where visible nodules were present in HT samples, these regions were prioritised for RNA extraction. Deep sequencing of feline thyroid RNA-seq libraries yielded a mean of 114,826,642 paired-end reads per library, with > 95% of reads mapping to the feline reference genome for all samples (Table S2). Principal component analysis (PCA) of normalised RNA-seq counts revealed separate clusters of samples according to FHT status, but not according to medical treatment (with thioureylene-based antithyroid drugs) status of HT cats (Fig. 1b, see also Table S1). Furthermore, we observed heterogeneity in the transcriptomes of thyroid samples from HT cats, with a minority of HT samples more closely associated with ET samples by PCA. Differential gene expression analysis, comparing all HT samples to ET samples, identified 948 differentially expressed genes (DEGs) with an adjusted p-value < 0.05, of which 484 had higher expression in HT samples compared to ET, and 464 had lower expression (Fig. 1c). Among the genes with higher expression in HT samples were several with well-defined roles in thyroid hormone synthesis, including *TPO*,* SLC5A5*,* DUOX1* and *TSHR* (Fig. S1a). Notably, expression of *SLC26A4*, which encodes the anion exchanger pendrin, important for transport of iodide across the thyrocyte apical membrane, was not detected in either ET or HT samples. We did, however, observe significantly higher expression of *SLC26A10*, predicted to also function as an anion transporter, in HT samples. *SLC26A7*, which has been reported to encode another thyroid-specific anion exchange protein^30^, was more highly expressed in the majority of HT samples relative to ET samples, but the difference was not significant (adjusted p-value > 0.05) (Fig. S1b). In addition, the gene encoding thyroglobulin, *TG*, was more highly expressed in the majority of HT samples but was only nominally significant in the differential gene expression analysis (Fig. S1c). Other genes with significantly higher expression in HT samples included inflammatory cytokines, such as *TNF* and *IL1B*, as well as growth factors *VEGFA* and *TGFB1*. DEGs with lower expression in HT samples included several involved in Wnt signalling, such as *WNT2*, *WNT4* and *SFRP2*, along with the growth factor receptors *EGFR*, *FGFR2* and *TGFBR2*. The full list of DEGs is available in Table S3. Enrichment analysis using Gene Ontology (GO) Biological Process feline gene sets revealed that genes with lower expression in HT samples were enriched for GO terms related to tissue growth and development, morphogenesis, and mesenchymal cell differentiation (Fig. 1d). Genes with higher expression in HT samples were enriched for several terms related to calcium ion transport, as well as regulation of cell morphogenesis and GTPase activity (Fig. 1e). All significantly enriched GO: BP terms are available in Table S4.

## Relationships between gene expression profile and histological grade

**Fig. 2:**
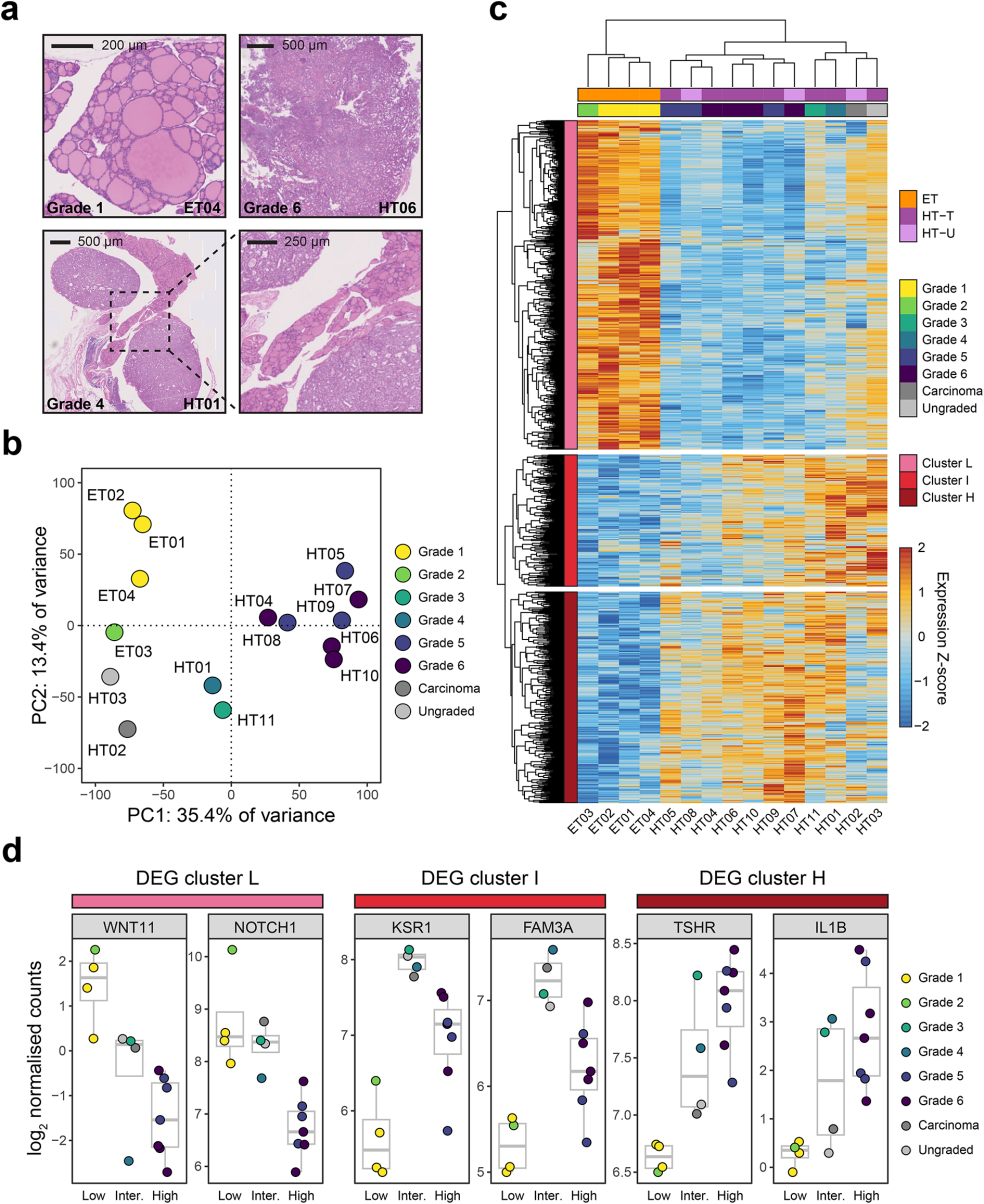
Relationship between histopathological grade and gene expression in thyroid samples. (**a**) Representative images of thyroid sections used for histopathological evaluation and grading. (**b**) As shown in Fig. 1a, samples are plotted based on scores from PCA and coloured by histopathological grade assigned in a blinded fashion following RNA-seq. (**c**) Heatmap showing mean-centred and scaled expression of 948 genes with differential expression (adjusted *p* < 0.05) between HT and ET samples. Unsupervised hierarchical clustering was used to group differentially expressed genes and samples based on expression of DEGs. (**d**) Selected example DEGs showing patterns of gene expression across samples for 3 DEG clusters identified in (**c**). Samples were grouped according to the sample clustering in (**c**): ‘Low’ – low histopathological grade (1 or 2), ‘Inter.’ - intermediate or undetermined histopathological grade (3 or 4, with one adenocarcinoma and one ungraded), and ‘High’ - high histopathological grade (5 or 6).

To aid the investigation of feline thyroid transcriptomic heterogeneity, the thyroid tissue samples used for RNA-seq underwent histopathological examination and grading in a blinded fashion by a board-certified veterinary pathologist (Fig. 2a). A summary of the results is available in Table S5. One sample, HT02, was identified as an adenocarcinoma upon histopathological examination. Insufficient tissue was available for the examination and grading of HT03. All other samples were assigned a grade from 1 to 6, according to the degree of hyperplasia and adenoma formation, with grade 1 assigned to normal thyroids with no evidence of hyperplasia (Fig. S2a), and grade 6 assigned to thyroids comprising a single large adenoma affecting > 80% of the gland (Fig. S2b) (see Methods). Epithelial hyperplasia and reduction in follicular lumen size was observed in samples with high histopathological grade, as well as morphological changes from flattened squamous epithelium with abundant colloid (grade 1) to predominantly columnar epithelium with limited visible colloid (grade 6) (Fig. S2c). Relabelling the RNA-seq samples by their assigned histopathological grade clearly explained the heterogeneity in HT samples observed during PCA of thyroid transcriptomes (Fig. 2b) and revealed that the cluster of seven HT samples with the largest separation from ET samples in the first principal component all showed signs of advanced adenomatous changes (i.e., grade 5 or grade 6). HT11, which was ambiguously assigned grade 3 or 6, was labelled as grade 3 based on its association with HT01 (grade 4) and separation from grade 5 and grade 6 samples during PCA. Unsupervised hierarchical clustering of DEGs and samples identified three clusters of DEGs, each characterised by high expression in one of three sample groups: cluster L - low-grade samples (grade 1 or grade 2), cluster I - intermediate-grade samples (grade 3 or grade 4, adenocarcinoma sample HT02 and ungraded HT03), and cluster H - high-grade samples (grade 5 or grade 6) (Fig. 2c). There was a clear trend in normalised expression levels for genes in each cluster relative to histopathological grade (Fig. 2d). Cluster L (low grade) contained all genes with lower expression in HT samples relative to ET samples, and therefore results of GO enrichment analysis with these DEGs were identical to those shown in Fig. 1c. For cluster I (intermediate grade), all significantly enriched GO: BP terms were related to GTPase activity and GTPase-mediated signalling (Fig S2d). Cluster H (high grade) DEGs, which had the highest expression in high-grade HT samples, were significantly enriched for terms related to calcium ion transport and response to toxic substance (Fig S2e). For many DEGs, we observed high variability in expression within the ‘intermediate’ sample group, which may have limited our ability to detect significant differences in gene expression between HT and ET samples.

## The FHT transcriptome reflects a hyperplastic and hypersecretory phenotype

**Fig. 3:**
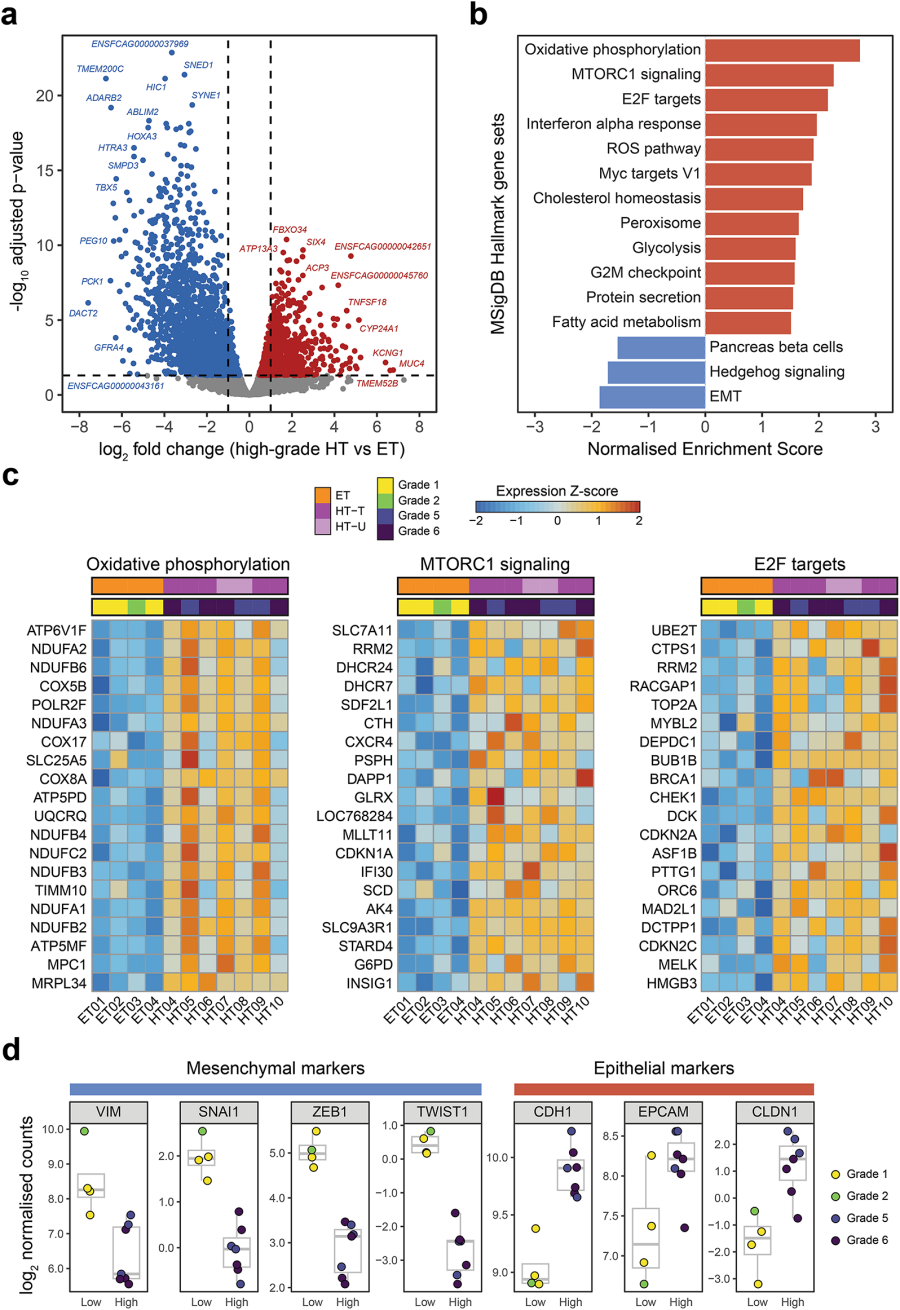
Differential expression analysis comparing HT samples with high histopathological grade and ET samples. (**a**) Volcano plot showing differentially expressed genes (adjusted *p* < 0.05) with higher expression in grade 1 or 2 ET samples (blue) or grade 5 or 6 HT samples (red). Grey points represent genes with no significant difference in expression between groups (adjusted *p* > 0.05). (**b**) Results of gene set enrichment analysis (GSEA) showing enriched MSigDB Hallmark feline gene sets for grade 5 or 6 HT samples (red) and grade 1 or 2 ET samples (blue). (**c**) Heatmaps showing normalised expression for the top 20 ranked genes for gene sets enriched in grade 5 or 6 HT samples. Normalised expression values for each gene are mean-centred and scaled across all samples. (**d**) Boxplots showing normalised expression of mesenchymal and epithelial marker genes in grade 1 or 2 ET samples and grade 5 or 6 HT samples.

Due to the heterogeneity of gene expression among intermediate-grade samples, a second differential gene expression analysis was performed, comparing only high-grade to low-grade samples (i.e., including grade 5 or 6 clinically HT versus grade 1 or 2 clinically ET, and excluding ungraded, carcinoma and grade 3 or 4 samples). This analysis identified 4,146 DEGs (adjusted p-value < 0.05), of which 1,909 had higher expression in high-grade HT samples compared to ET samples, and 2,237 had lower expression (Fig. 3a). The full list of DEGs is available in Table S6. The majority of genes in clusters L and H from the original analysis (88.1% and 81.9%, respectively) were also differentially expressed in the analysis of high versus low grade samples, while only 46.2% of genes in cluster I were significant in both analyses. We observed stronger negative fold changes in the second analysis for genes in cluster L and weaker positive fold changes for genes in cluster I (Fig. S3a). This confirmed that the intermediate-grade sample group was influencing fold change estimates in the first analysis. GO enrichment analysis using the DEGs from the second analysis revealed that genes with higher expression in ET samples were enriched for similar terms as in the first analysis, related to tissue development and morphogenesis (Fig. S3c). For genes with higher expression in high-grade HT samples, enriched terms were strikingly different to those for all HT samples in the original analysis and were mostly related to mitochondrial respiration (Fig. S3b). As an additional investigation of pathway enrichment, Gene Set Enrichment Analysis (GSEA) was performed using normalised RNA-seq counts from high-grade HT and ET samples to test for enrichment of 50 MSigDB Hallmark feline gene sets. Of these 50 gene sets, 12 were significantly enriched for genes with higher expression in high-grade HT samples, and 3 were significantly enriched for genes with higher expression in ET samples (FDR < 0.05) (Fig. 3b). For genes with higher expression in high-grade HT samples, the strongest enrichment was observed for the oxidative phosphorylation gene set (Fig. S3d). Several metabolic gene sets (e.g. MTORC1 signalling, cholesterol homeostasis, glycolysis, fatty acid metabolism) were also significantly enriched, as well as gene sets related to the cell cycle (including targets of the E2F family and Myc transcription factors), redox homeostasis and protein secretion. Although individual genes were heterogeneously expressed among high-grade HT samples, GSEA revealed clear signatures of increased expression for functionally related genes in all grade 5 or 6 HT thyroid samples relative to ET samples (Fig. 3c). In contrast, for genes with higher expression in ET samples, the strongest enrichment was observed for the epithelial-mesenchymal transition (EMT) gene set (Fig. S3e). Several genes encoding key markers and transcriptional regulators of EMT had significantly lower expression in high-grade HT samples compared to ET samples, such as *VIM*, *SNAI1*, *SNAI2*, *ZEB1/2* and *TWIST1/2*, while genes encoding epithelial cell markers, such as *CDH1*, *EPCAM* and *CLDN1*, had significantly higher expression in high-grade HT samples (Fig. 3d). When expression of these genes was compared across the full cohort of HT samples included in the original differential gene expression analysis, they were expressed in the samples with histopathological grades 3 and 4 (HT11 and HT01, respectively) at levels between high-grade HT and ET cats. Interestingly, expression of these markers in HT02 and HT03 (of which HT02 was confirmed as adenocarcinoma) differed in the opposite direction to the grade 3 and grade 4 samples (Fig. S3f), suggesting that EMT may be differentially regulated in benign and malignant thyroid nodules.

## Network analysis highlights additional pathways dysregulated in FHT

**Fig. 4:**
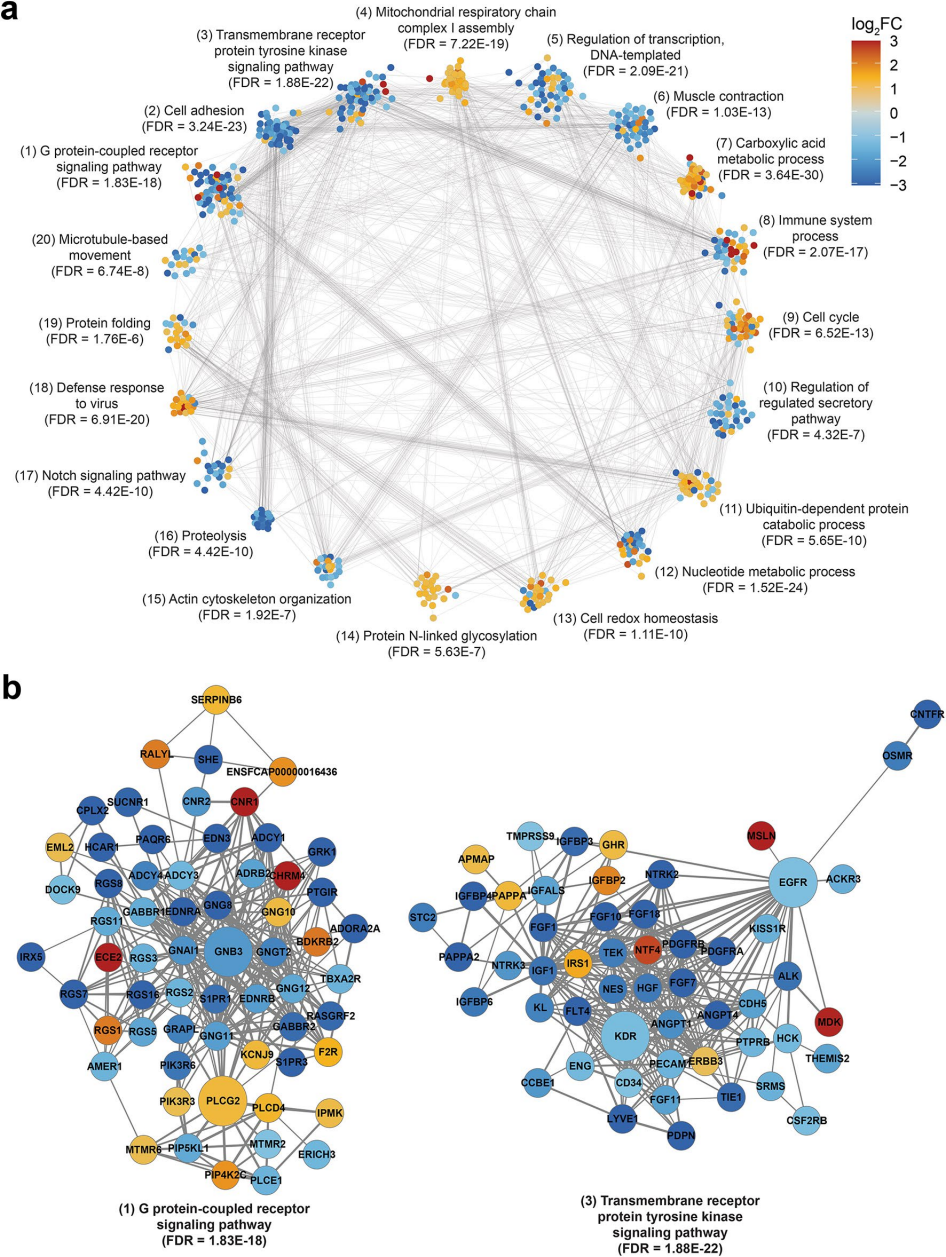
Protein-protein interaction (PPI) networks for DEGs between high-grade HT and ET samples. (**a**) 20 largest MCL gene clusters from a PPI network of 2,290 DEGs (with absolute fold change > 2). Each node represents a DEG and is coloured by log_2_ fold change between high-grade HT and ET samples. Edges (grey lines) connect nodes with evidence for PPI in the STRING database. Labels show the most enriched GO: BP for genes in each cluster, along with FDR for enrichment. (**b**) Zoomed view of clusters 1 (left) and 3 (right) from (**a**). Nodes are labelled with the associated protein product and coloured by log_2_ fold change as in (**a**). Edge width is proportional to the STRING association score (minimum 0.4). Larger nodes represent hub genes (identified as highly connected in the main network).

To further explore relationships between DEGs associated with high-grade FHT, a protein-protein interaction network was constructed from DEGs between high-grade HT and ET samples with an absolute fold change greater than two. From the 2,831 DEGs used as input, a single network comprising 2,290 interacting proteins was retained for further analysis (Fig. S4a). The most highly connected genes (i.e. high node degree) in the network tended to have higher values for BetweennessCentrality (a measure of how often a node lies on the shortest path between two other nodes), and included several with well-studied roles in cell growth and survival pathways, such as *EGF*, *EGFR*, *TNF* and *PLCG2*. Other highly connected genes were less clearly related to thyroid development and function, such as *LRRK2* and *TTN* (Fig. S4b). We used the Markov Cluster (MCL) algorithm to identify subnetworks of highly interconnected nodes within the main network, which resulted in 130 clusters of at least 5 connected nodes (1,453 nodes in total). Functional enrichment analysis of clustered DEGs was used to annotate each cluster with the most strongly enriched GO: BP term (i.e. lowest FDR) (Table S7), and the enriched terms for the largest clusters included several related to tissue growth, development, mitochondrial respiration and redox homeostasis, and overlapped with the results of the previous GSEA and GO analyses (Fig. 4a). Cluster 1, the largest cluster containing 62 nodes and 283 edges, included a number of DEGs involved in signalling through G protein-coupled receptors (GPCRs), such as *GNB3* and *GNAI1* (Fig. 4b). The majority of genes in cluster 1 had lower expression in high-grade HT samples compared to ET samples, including multiple isoforms of adenylate cyclase (*ADCY1*, *ADCY3*, *ADCY4*), suggesting downregulation of genes potentially involved in G_s_-coupled GPCR signalling in FHT. The most highly expressed adenylate cyclase isoforms in the thyroid RNA-seq samples, *ADCY6* and *ADCY9*, were not differentially expressed between high-grade HT and ET samples, indicating that at least some G_s_α-adenylate cyclase signalling remains intact in FHT. In cluster 12, other genes directly involved in G_s_-coupled GPCR signalling encoding 3’,5’-cyclic phosphodiesterases that hydrolyse cAMP, such as *PDE2A*, *PDE3A* and *PDE7B*, had significantly lower expression in high-grade HT samples compared to ET samples.

Cluster 3, containing 53 nodes and 271 edges, included DEGs involved in signalling by receptor tyrosine kinases (RTKs), such as *KDR* and *EGFR* (Fig. 4b). As for cluster 1, the majority of these DEGs had lower expression in high-grade HT samples compared to ET samples, indicating that signalling by several RTKs is downregulated in hyperplastic thyroid tissue. We observed higher expression in high-grade HT samples for the majority of DEGs in clusters related to mitochondrial respiration and fatty acid metabolism (Fig. S4c). We also observed lower expression in high-grade HT samples for the majority of DEGs in a number of smaller clusters involved in signalling pathways known to regulate EMT, such as Notch, Wnt and Hedgehog/Smoothened^31–33^, as well as pathways involved in regulating tissue growth and development, such as ephrin receptor and semaphorin-plexin signalling, which have also been linked to EMT^34,35^ (Fig. S4d). Also of note, cluster 8 included several DEGs encoding inflammatory cytokines, such as *TNF*, *IL1B* and *CXCL8*, which had higher expression in high-grade HT samples compared to ET samples, and cluster 18 DEGs also had mostly higher expression in high-grade HT samples, and predominantly related to interferon signalling, suggesting that inflammatory pathways are associated with FHT (Fig. S4e).

### Activating mutations in GNAS (G_s_α) are associated with FHT

**Fig. 5:**
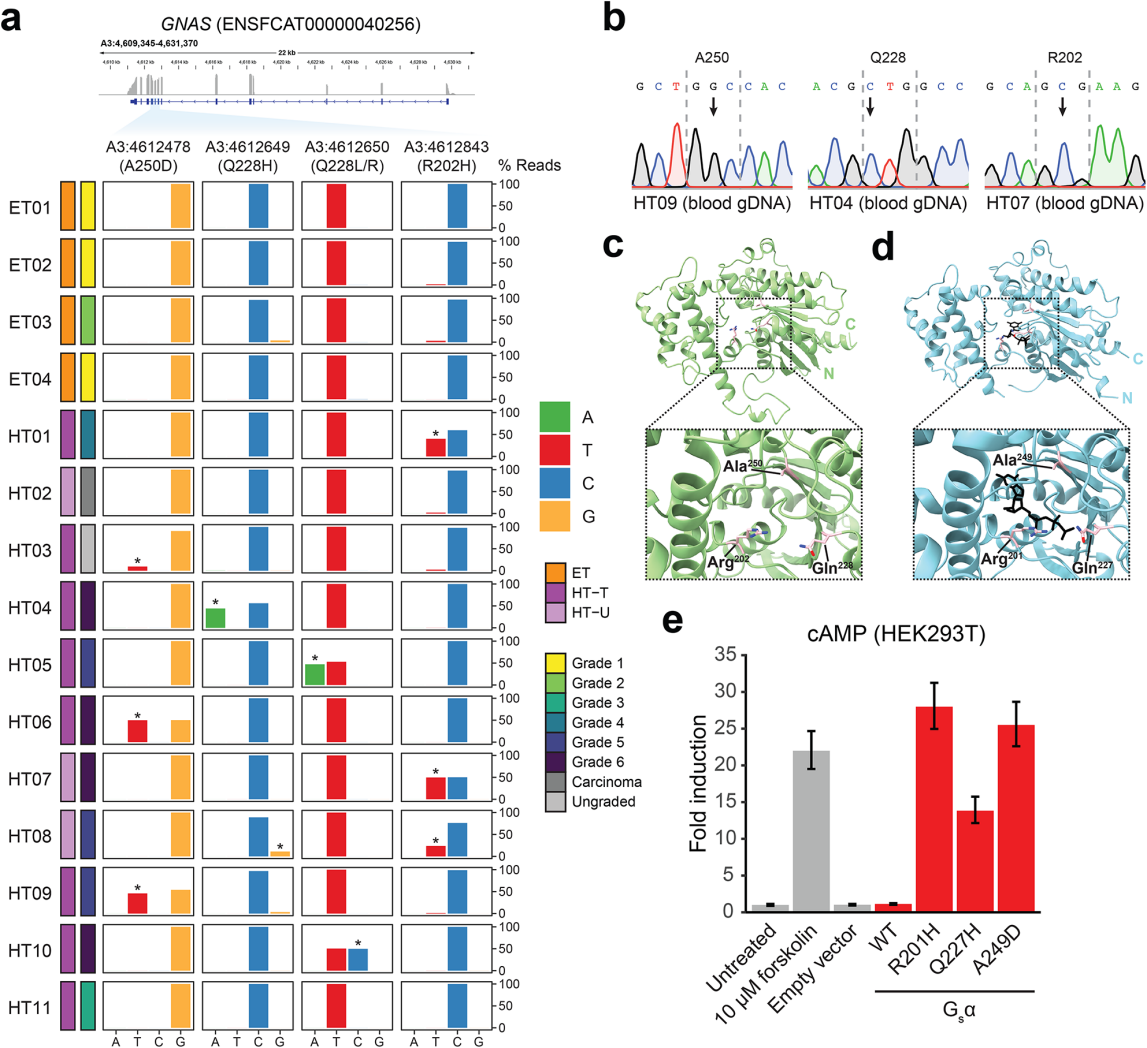
Detection of activating G_s_α mutations in RNA-seq reads from HT samples. (**a**) Nucleotide frequencies in mapped RNA-seq reads at variant positions in *GNAS* (ENSFCAT00000040256). Alternative alleles called by GATK are marked with *. (**b**) Sanger sequencing results for gDNA from representative HT cats with thyroid G_s_α mutations detected by RNA-seq. Sequences are shown for the + strand of the feline genome, and positions of G_s_α codons on the - strand are marked with grey dashed lines. Variant positions in the related thyroid sample are marked with a black arrow. (**c**) AlphaFold predicted structure for feline G_s_α (ENSFCAG00000034898, UniProt A0A2I2U232) with a zoomed view of the residues (labelled and sidechains shown in pink) altered by the variants shown in (**a**). (**d**) Crystal structure of *Bos taurus* G_s_α (PDB: 1AZT) with bound GTPγS (black). The same residues as for *Felis catus* G_s_α shown in (**c**) are highlighted (labelled and sidechains shown in pink). (**e**) Representative cAMP assay results for HEK293T cells transfected with wild-type (WT) and mutant G_s_α. Fold induction is relative to untreated cells. Values shown are means of *n* = 4 wells of a 96-well plate. Error bars show 95% confidence intervals. The assay was performed a total of 3 times, from 3 independent transfections.

In order to investigate the potential role of mutations in TSHR and G_s_α in our dataset, we used GATK to detect and genotype variants in RNA-seq reads from HT and ET cats. Focused analysis of variants in exon 10 of *TSHR*, encoding the transmembrane and intracellular domains of TSHR and the focus of previous efforts to identify FHT-associated mutations, identified three missense variants, of which two had been identified in a previous study^23^ (Fig. S5a). One of these variants (S6) was detected in a single HT cat (HT02 – adenocarcinoma), and two ET cats (ET01 – grade 1, ET04 – grade 1). The other previously identified missense variant (8a/8b) was detected in only two HT cats (HT08 – grade 5, HT11 – grade 3). The third missense variant, novel to this study, resulted in a p.M727V substitution in the intracellular domain of TSHR and was detected in three HT cats (HT07 – grade 6, HT09 – grade 5, HT11 – grade 3). Although we could not rule out the involvement of *TSHR* variants in FHT pathogenesis, they were not strongly associated with FHT in our study. In contrast, in exons 8–10 of *GNAS*, encoding G_s_α, we detected missense variants in 9/11 thyroid samples from HT cats, and none of these variants were detected by GATK in thyroid samples from ET cats (Fig. 5a). Quantification of mapped reads containing alternate alleles at each site revealed low frequency *GNAS* variants in a number of samples called as homozygous for the reference allele by GATK (Table S8). Interestingly, the RNA-seq sample from ET03, which was assigned grade 2 by histopathological evaluation indicating nodular hyperplasia affecting less than 50% of the gland (see Methods), included reads containing alternate alleles for the p.R202H and p.Q228H variants at 3.10% and 3.82%, respectively, suggesting subclinical hyperthyroidism in this cat. At least one missense variant in *GNAS* exons 8–10 was detected in all high-grade HT samples, and substitutions at two of the three amino acid residues affected, p.R202H and p.Q228H, have been previously reported to result in constitutive activation of G_s_α and endocrine tumours in humans^36^, but the impact of the p.A250D substitution is unknown. The two HT cats without missense *GNAS* variants included HT02, which we identified as a case of adenocarcinoma by histopathology, and HT11, which was assigned grade 3 during histopathological evaluation. Blood samples were available for 11/15 cats included in the study (*n* = 4 ET, *n* = 7 HT), and Sanger sequencing of *GNAS* exons 8–10 in genomic DNA isolated from peripheral blood cells confirmed the absence of germline *GNAS* variants in these cats (Fig. 5b). We also searched for the feline thyroid *GNAS* variants in the 99 Lives Cat Genome Sequencing database^37^, but none were present, suggesting that the *GNAS* variants detected by RNA-seq are acquired mutations restricted to the thyroid.

To evaluate the potential functional impact of the three mutated amino acid residues, we generated a structural model for feline G_s_α using AlphaFold^38^, and observed that all three residues were proximal to the GTP-binding pocket in the GTPase domain (Fig. 5c). The amino acid sequence of G_s_α is highly conserved, and wild type feline G_s_α differs from both human and bovine G_s_α by insertion of a single serine at position 87 (Fig. S5b). Comparison with the previously published crystal structure of bovine G_s_α (PDB: 1AZT), with hydrolysis-resistant GTPγS bound, showed a similar arrangement of the mutated residues around the GTP-binding pocket (Fig. 5d). To determine the functional impact of the novel p.A250D G_s_α mutation detected in 3 of the HT cats in this study, we generated plasmids for overexpression of human G_s_α with equivalent mutations to those detected in HT cats (p.R202H > R201H, p.Q228H > Q227H and p.A250D > A249D, Fig. S5b). The resulting constructs were transfected into HEK293T cells and cAMP levels were determined by luciferase assay following treatment with the phosphodiesterase inhibitors 3-isobutyl-1-methylxanthine (IBMX) and Ro-20-1724. A strong increase in intracellular cAMP was observed for cells transfected with the three mutant G_s_α constructs, whereas cells transfected with wild-type (WT) G_s_α had cAMP levels similar to untransfected and empty vector controls (Fig. 5e). The increase was similar to that observed following treatment of untransfected cells with forskolin, which activates adenylate cyclase. Taken together, these results show that the G_s_α variants detected in feline thyroids in this study are acquired mutations, and that these mutations result in constitutive G_s_α activation and elevated intracellular cAMP.

## Discussion

Spontaneous feline hyperthyroidism (FHT) is highly prevalent in the domestic cat population and shares similarities to toxic multi-nodular (TMG) goitre in humans, yet its etiopathogenesis remains poorly understood^13^. Persistent activation of adenylate cyclase downstream of G_s_ may be involved in FHT, since TSH is chronically suppressed in HT cats, and expression of genes relating to thyroid hormone synthesis is mainly regulated by G_s_-coupled TSHR activation. In this study, we sought to identify critical genes and mechanisms in FHT by performing transcriptomic and mutation analysis of thyroid tissue from clinically HT and ET cats. Here, as well as revealing molecular pathways dysregulated in FHT, we detected somatic mutations in feline *GNAS*, encoding the G protein subunit G_s_α. Not only were these mutations strongly associated with FHT, but they also resulted in constitutive activation of G_s_α and elevated cAMP in vitro, consistent with a causal role in FHT.

Amongst the 948 genes differentially expressed between HT and ET samples, higher expression of genes involved in thyroid hormone synthesis such as *TPO*, *SLC5A5*, *DUOX1* and *TSHR* was observed in HT samples (Fig. S1a). Unexpectedly, we were unable to detect expression of *SLC26A4*, encoding the iodide transporter pendrin, critical for thyroid hormone synthesis in humans. However, a predicted alternative iodide transporter *SLC26A10* showed significantly increased expression in HT thyroids, along with another transporter *SLC26A7*, known to contribute to iodide efflux from thyrocytes^30^, which showed a trend towards higher expression (Fig. S1b).

We also detected differential expression of genes involved in calcium ion transport, GTPase activity and redox homeostasis (Fig. 1d). Calcium homeostasis is crucial for myriad cellular processes, and in the thyroid, phospholipase C (PLC) activation by G_q_-coupled TSHR signalling promotes the IP_3_-dependent release of Ca^2+^ from the ER, and negatively regulates TSHR signalling via G_s_^18^. Conversely, elevated intracellular cAMP modulates Ca^2+^ transport in FRTL-5 rat thyrocytes^39,40^. Increased expression of Ca^2+^ transport genes in FHT may, therefore, represent a negative feedback mechanism for moderation of excessive G_s_-mediated intracellular cAMP production.

The genes with lower expression in HT samples were significantly enriched for GO terms related to tissue development and morphogenesis (Fig. 1e), with expression of the growth factor receptor *EGFR* notably reduced in HT samples. The role of EGF and its receptor in thyroid epithelial cell growth and function remains uncertain, but extensive crosstalk between GPCR and receptor tyrosine kinase (RTK) signalling pathways has been documented^41^. Moreover, TSHR has been shown to transactivate EGFR in a ligand-independent way during TSH-stimulated signalling, while the effect of EGF is antagonistic to that of TSH, with respect to expression of thyroid hormone synthesis genes^42^. Lower expression of *EGFR* in HT samples may therefore reflect another example of a negative feedback mechanism limiting the potential for further stimulation of growth in the presence of constitutive TSHR signalling.

Finally, several genes involved in TGF-β signalling were differentially expressed in HT thyroid tissue, including higher expression of *TGFB1* itself, but also higher expression of inhibitory *SMAD7* and lower expression of ligand binding *TGFBR2* and the coreceptor *TGFBR3*. TGF-β is known to play an important role in restricting thyroid epithelial cell proliferation indicating that, despite higher expression of *TGFB1*, sensitivity to TGF-β is reduced in FHT, as has been observed for thyroid tumours in humans^43,44^.

The transcriptomic heterogeneity of HT samples was largely explained by histopathological grading of the tissue used for RNA-seq, where gene expression was correlated with degree of hyperplasia (Fig. 2). Whilst care was taken during dissection prior to RNA extraction to include only nodular tissue, it is possible that the thyroid transcriptome captured for some cats also contains surrounding non-hyperplastic tissue. However, the concurrent histopathological examination of the tissues from which RNA was extracted indicated that the hyperplastic tissue, where present, was spread throughout the thyroid in high-grade samples (Fig. S2). The majority of HT cats included in the study were treated with anti-thyroid drugs, such as methimazole, which potently inhibits thyroid peroxidase and effectively limits the release of thyroid hormone, however there were no clear differences in gene expression between treated and untreated HT cats. This reflects the fact that thioureylene-based therapies address only the excess thyroid hormone secretion arising as a consequence of functional thyroid hyperplasia and not the underlying pathogenesis of disease. We identified one case of adenocarcinoma (HT02), and one sample could not be graded (HT03) due to lack of available fixed tissue, although based on results of PCA and unsupervised clustering of DEGs this was suspected to be another case of adenocarcinoma. HT02 and HT03 appeared to express lower levels of thyroid hormone synthesis genes than other HT samples of grade 3 and above. This was particularly evident for *TG*, encoding the thyroid hormone precursor thyroglobulin, of which HT02 and HT03 had the lowest expression among HT samples.

This study is not powered to detect clear differences between the underlying biology of thyroid adenoma and carcinoma. Therefore, further work is required to determine whether any genes will show consistent differential expression between small thyroid adenocarcinomas, larger thyroid adenocarcinomas and thyroid adenomas^45^. Consequently, subsequent analysis selectively compared only high-grade HT samples and low-grade ET samples, facilitating more reliable distinction of molecular pathways influencing formation of adenoma in advanced FHT. This focused analysis revealed a hyperplastic and hypersecretory phenotype in high-grade HT samples, and as expected, indicated increased metabolic demand (Fig. 3).

Multiple gene sets involved in progression through the cell cycle were significantly enriched in high grade HT samples, including ‘E2F targets’ and ‘Myc targets V1’. Although *MYC* was not differentially expressed in our study, c-myc expression has been associated with hyperplastic thyroid epithelial cells in Graves’ Disease in humans^46^, and c-myc mRNA is transiently increased in FRTL-5 cells treated with either TSH or cAMP, prior to an increase in cell proliferation^47^. TSH stimulation activates MAPK in thyrocytes through both PKA-dependent and independent mechanisms^48,49^. MAPK activation is also downstream of growth factor receptors, however we observed generally lower expression of a PPI network comprising genes encoding growth factors and their receptors, including *IGF1* and *EGFR* in HT samples (Fig. 4). The gene set ‘MTORC1 signalling’ was also significantly enriched in HT samples, and TSH is known to promote PKA-dependent activation of the PI3K/Akt pathway, and thereby mTORC1, in thyroid epithelial cells^50^. In addition, binding of TSH to TSHR and activation of G_s_α releases Gβγ, which can also activate PI3K/Akt^51^.

This study is the first to report a signature of mesenchymal-epithelial transition (MET) in feline hyperthyroid tissue, with several key regulators of this process among the DEGs identified in the high-grade HT versus ET analysis. The reverse process, epithelial-mesenchymal transition (EMT), has previously been associated with thyrocyte morphology and function in PCCL3 cells, where conditional inhibition of DNA binding by TTF-1 (NKX2-1) resulted in increased expression of vimentin, decreased expression of E-cadherin, and morphological changes consistent with EMT^52^. Subsequent removal of this inhibition restored epithelial cell morphology. Although we detected no significant difference in expression of *NKX2-1* between high-grade HT and ET samples, cAMP-dependent phosphorylation of TTF-1 by PKA has been shown to increase its transcriptional activity in thyrocytes^53^. Consistent with that observation, we detected lower expression of mesenchymal markers, including *VIM*, and higher expression of epithelial markers, such as *CDH1* (encoding E-cadherin), in high-grade HT samples, implying a potential role for cAMP-dependent phosphorylation of TTF-1 in FHT.

A limitation of this study is low sample number, particularly in the analysis comparing high-grade HT samples to ET samples. However, the samples included were from extensively phenotyped cases with detailed clinical histories. As the first study of the thyroid transcriptome in FHT, these results provide a solid foundation for understanding the molecular pathogenesis of hyperthyroidism in cats. Future studies in expanded cohorts, including a larger number of intermediate-grade (3 or 4) HT samples, will further clarify the relationship between FHT progression and disease severity.

Taken together, we hypothesised that differences in gene expression between HT and ET samples were attributable to constitutive signalling through TSHR/G_s_α/adenylate cyclase. This has been previously described for some cases of TMG in humans, where activating mutations in TSHR or G_s_α result in chronically elevated intracellular cAMP in thyrocytes and promote hyperplasia and increased thyroid hormone synthesis^21^. By calling variants in our RNA-seq reads from HT and ET samples, we examined mutations in several candidate genes for their association with the FHT phenotype and potential impact on gene function (Figs. 5, S5). Critically, the absence of the identified mutations in extrathyroidal genomic DNA from HT cats included in the RNA-seq experiment indicated that these were acquired mutations in the thyroid, a finding supported by the absence of the mutations from the largest available database of feline genomic variation, the Feline 99 Lives database^37^.

Previous candidate gene studies have focused on mutations in TSHR and in G_s_α, encoded by *GNAS*. Watson et al.^23^ reported mutations in exon 10 of TSHR, encoding the transmembrane and intracellular domains, in thyroid nodules from 28/50 HT cats. In the present study, however, variants predicted to alter the structure of TSHR were found in only a minority of HT cats and were also seen in ET cats, suggesting that they are not sufficient on their own to cause hyperthyroidism. Two of the TSHR mutations identified in this study had previously been reported by Watson et al., although crucially no ET cats were included in that study. One of the 3 TSHR mutations, S8a/8b (p.D632Y/H), was exclusively found in HT cats in the present study, and one was a novel mutation of unknown significance, p.M727V, located in the intracellular domain of TSHR in 3 HT cats (Fig. S5a).

In addition to the identified TSHR mutations, a strong association was observed between FHT and missense variants altering 3 different residues in the region of *GNAS* encoding the Ras-like GTPase domain of G_s_α. Peeters et al. detected G_s_α mutations in 4/10 HT cats at two of the sites identified as variant in the present study (p.R202H and p.Q228H)^24^. The third mutation identified here has not previously been reported and could not have been detected in the Peeters et al. study because the site was not included in the PCR amplicon (V160 to D241 in feline *GNAS* cDNA).

Functional investigation revealed that all 3 acquired *GNAS* mutations influence cAMP production in vitro, in a manner consistent with constitutive G_s_α activation, supporting a causal role in FHT. Strikingly, only 2 HT cats in our study lacked G_s_α mutations, HT02 and HT11, of which HT02 was confirmed to be a case of adenocarcinoma, and HT11 was grade 3, the lowest grade of the clinically HT cats included in the RNA-seq. Notably, however, HT11 thyroid tissue carried both p.D632Y/H and p.M727V TSHR mutations, and therefore the potential involvement of TSHR mutations in some cases of FHT warrants further study.

G_s_α is ubiquitously expressed and is involved in signaling through a number of different GPCRs, as well as TSHR. As such, therapeutic inhibition of constitutively active G_s_α in the thyroid may have unintended consequences. However, a recent study identified a G_q_-biased TSHR agonist, MSq1, which inhibited TSH-induced PKA-dependent proliferation of thyrocytes in vitro^18^. Future work examining the effect of such molecules on thyroid epithelial cells expressing G_s_α with the activating mutations detected in this study may lead to viable thyroid-specific therapies reducing thyroid hyperplasia in FHT.

In conclusion, the feline species offers a novel, high prevalence, spontaneous model of human TMG. The approach described here could also be applied to evaluation of other recognised feline endocrinopathies that are characterised by functional adenomatous changes, such as hyperaldosteronism and hypersomatotropism. Taken together, the results of this study indicate a critical role for constitutive G_s_α activation in FHT, which persists despite medical therapy. Somatic mutations in affected thyroids in *TSHR* and *GNAS* are associated with FHT status, with the constitutive G_s_α activation resulting from *GNAS* mutations supporting the causality of these mutations in disease pathogenesis. Future work focused on spatial and single-cell transcriptomic data, as well as in situ detection of somatic mutations within the thyroid will clarify further the relationship between nodular change, hyperplasia and hyperfunction in FHT, helping to identify novel therapeutic or preventative targets.

## Materials and methods

### Thyroid tissue collection

Thyroid tissue was obtained by surgical therapeutic thyroidectomy or by post-mortem tissue collection from cats attending one of two first opinion practices in London (Beaumont Sainsbury Animal Hospital and People’s Dispensary for Sick Animals), and from cats euthanised utilising intravenous pentobarbital for clinical reasons unrelated to this study at Queen Mother Hospital for Small Animals, Royal Veterinary College (RVC). Hyperthyroidism was diagnosed based on appropriate clinical signs and elevated plasma T4 concentration (*n* = 10) or high-normal plasma T4 in combination with undetectable TSH (*n* = 1). Euthyroid cats had plasma T4 concentration within the laboratory reference interval, non-suppressed TSH and no reported signs of thyroid disease. The majority of HT cats were treated with thioureylene-based antithyroid drugs, and the treatment status and duration of all cats included in the study is available in Table S1. Thyroids were stored in RNAlater (Sigma-Aldrich) at -80^o^C for a maximum of 80 months prior to RNA extraction. The RVC Ethics and Welfare Committee approved data and tissue collection and storage from cats included in this study (reference no. CRERB2013-1258) and informed consent for research use of blood and tissue that was surplus to clinical requirements was obtained from the cats’ owners. This study did not influence the veterinary care of any patient and all samples were collected and archived in accordance with relevant regulations. A summary of the clinical information for the cats included in the study is available in Table S1.

## Histopathological evaluation of thyroid tissue

Sections from formalin-fixed thyroid tissue were prepared and stained with haematoxylin-eosin. Histological examination was performed in a blinded fashion as described previously^54^, and samples were graded for hyperplasia and adenomatous changes as follows: grade 1 – normal gland without evidence of nodular hyperplasia, grade 2 – nodular hyperplasia affecting < 50% of the gland, grade 3 – nodular hyperplasia affecting > 50% of the gland, grade 4 – nodular hyperplasia including at least one partially encapsulated hyperplastic node, grade 5 – small adenoma(s) on a background of nodular hyperplasia and grade 6 – single large adenoma affecting > 80% of the gland.

### RNA extraction

A 3–4 mm^3^ sample of each thyroid was dissected with scissors and washed with PBS prior to lysis in TRIzol reagent (Invitrogen). Where a distinct nodule or nodules were present in hyperthyroid cats, this area of tissue was prioritised for RNA-Seq. Phenol-chloroform extraction was used to isolate nucleic acids from the lysate, and RNA was purified from the resulting aqueous phase using a RNeasy Micro kit (Qiagen) with on-column DNase treatment to digest genomic DNA. Pure RNA was eluted with RNase-free water, and RNA integrity and concentration were determined using a Tapestation 4200 (Agilent) and Nanodrop ND-1000 (Thermo Scientific) respectively. Due to low A260/230 ratios for some samples, all RNA samples were cleaned up using an RNA Clean & Concentrator-25 kit (Zymo Research).

### RNA-seq library preparation and sequencing

RNA-seq libraries were prepared from total RNA by Oxford Genomics Centre (Wellcome Centre for Human Genetics, University of Oxford). Due to the presence of degradation in most samples (RIN < 8), libraries were prepared following ribodepletion with the NEBNext rRNA Depletion Kit v2 (NEB) to remove abundant rRNA species. Stranded libraries were generated using a TruSeq Stranded Total RNA kit (Illumina) and sequenced in two batches as either 75 or 150 bp paired-end reads on a HiSeq4000 (Illumina) or NovaSeq6000 (Illumina) instrument, respectively. High-depth sequencing resulted in a minimum of 80 million paired-end reads for each library, with the aim of mitigating the impact of RNA degradation on quantification of genes with low expression.

### RNA-seq data processing and analysis

Raw read quality was assessed using FastQC. Adapters were trimmed from raw reads using PEAT^55^. Trimmed reads were aligned to the felCat9 reference genome (GCA_000181355.4) using STAR^56^, and resulting alignments were filtered with Samtools^57^ to retain only properly paired, uniquely mapped reads. Gene-level counts were obtained using the featureCounts function from the Rsubread package^58^with felCat9 gene annotations from Ensembl. Counts were normalised using the trimmed mean of M-values (TMM) method in edgeR^59,60^ and filtered to remove genes with low expression. Differential expression between hyperthyroid and euthyroid samples was then tested using edgeR with inclusion of a coefficient for sample batch in the generalised linear model. Genes were considered differentially expressed if the (Benjamini-Hochberg) adjusted p-value was < 0.05. Principal component analysis was performed on normalised counts using the prcomp function in R, and unsupervised hierarchical clustering of differentially expressed genes and samples was performed using the default clustering method of the pheatmap package (https://github.com/raivokolde/pheatmap). Gene expression was plotted as log_2_-transformed TMM-normalised counts per million mapped reads (CPM).

### Pathway enrichment analysis

GO enrichment analysis was performed using clusterProfiler^61^ with annotated feline gene sets available from the msigdb package in R/Bioconductor. GO Biological Process gene sets with a FDR-adjusted p-value < 0.05 were considered significantly enriched. For GSEA, feline Hallmark gene sets, also retrieved using the mSigDB package in R, were used with TMM-normalised RNA-seq counts from edgeR in the desktop GSEA application from the Broad institute^62,63^ (v4.3.2). Hallmark gene sets with a FDR-adjusted p-value < 0.05 were considered significant.

### Network analysis

A protein-protein interaction network was constructed from 2,831 DEGs (adjusted p-value < 0.05) with an absolute fold change > 2 in the high-grade HT vs. ET differential expression analysis using the stringApp plugin (v2.0.3) in Cytoscape (v3.10.1), with species set to ‘Felis catus’. The confidence (score) cutoff was set to 0.4, with maximum additional interactors set to 0 (the default). The largest subnetwork, containing 2,290 connected nodes, was clustered with the ‘MCL Cluster’ algorithm from the clusterMaker2 plugin (v2.3.4), and each cluster was annotated with the most strongly enriched GO: BP term for the nodes in that cluster.

### RNA-seq variant calling

Reads were re-mapped to felCat9 using the 2-pass alignment mode in STAR^56^. Duplicate reads were marked with Picard^64^ and marked BAM alignments were pre-processed according to the GATK4 best practices workflow for variant calling from RNA-seq data^65^. Briefly, the SplitNCigarReads, BaseRecalibrator, ApplyBQSR and HaplotypeCaller tools were used to obtain preliminary variant calls for mapped RNA-seq reads from each sample. Variants were hard-filtered using parameters recommended by GATK: --window 35, --cluster 3, FS > 30.0 and QD < 2.0. Resulting VCFs were merged and annotated with snpEff^66^. Counts for reads containing different bases mapped to variant positions in exons 8–10 of *GNAS* (ENSFCAT00000040256) were obtained using Samtools mpileup. Mutant residues in the Alphafold2 prediction for the translation product of the major *Felis catus* G_s_α transcript expressed in the RNA-seq samples (Ensembl: ENSFCAT00000040256, UniProt: A0A2I2U232) and the GTP-bound G_s_α protein from *Bos taurus* (PDB: 1AZT) were visualised using ChimeraX^67^.

### Genotyping of feline blood samples

Genomic DNA was extracted from 100 µl EDTA-blood using a DNeasy blood and tissue kit (Qiagen) with RNase digestion. The region containing exons 8–10 of *GNAS* (ENSFCAT00000040256) was amplified in a 25 µl PCR reaction containing 50 ng gDNA, 12.5 µl 2X Biomix Red (Bioline), and the following primers at 800 nM: 5’-TCCAAGCTCGTCAGGATCTC-3’ and 5’-GAGAGGGTGCAGGTTTAAGGT-3’. For each sample, 10 µl PCR product was cleaned up using ExoSAP-IT Express reagent (ThermoFisher Scientific) according to the manufacturer’s instructions, and then subjected to Sanger sequencing (Source Bioscience).

### Gsα cloning and site-directed mutagenesis

The wild-type (WT) sequence of human G_s_α was amplified from pEM705 Gs^68^ (a gift from Robert Lucas, Addgene plasmid #109350; http://n2t.net/addgene:109350; RRID: Addgene_109350) in a PCR reaction containing 1 ng pEM705 Gs, 0.02 U/µl Q5 High-Fidelity DNA Polymerase (NEB), 200 µM dNTPs (Bioline) and the following primers at 500 nM: 5’-TATACTCGAGACCATGGGCTGCCTCGG and 5’-ATTAAAGCTTTCAGAGCAGCTCGTACTGAC. Thermocycling conditions were according to the recommendations of the polymerase supplier, with an annealing temperature of 67 °C and an extension time of 40 s, for 30 cycles. The PCR product was cloned into pcDNA3.1(-) (Invitrogen) by restriction digestion of both insert and vector using XhoI (NEB) and HindIII (NEB) followed by ligation with T4 DNA ligase (NEB). Gsα mutants were generated by site-directed mutagenesis of the pcDNA3.1-Gs construct using the Q5 Site-Directed Mutagenesis Kit (NEB). For each of the Gsα mutations detected in HT cats, the equivalent mutations in human Gsα were generated using the following mutagenic primer pairs (mutant codons lower case, nucleotide substitutions in italic): R201H − 5’-GCTTCGCTGCc*a*tGTCCTGACTT and 5’-AGGTCCTGATCGCTCGG (T_a_ = 68°C), Q227H − 5’-CGTGGGTGGCca*t*CGCGATGAAC and 5’-TCAAACATGTGGAAGTTGACTTTGTCCAC (T_a_ = 70 °C), A249D − 5’-CTTCGTGGTGg*a*cAGCAGCAGCT and 5’-ATGATGGCAGTCACATCGTTGAAGCAC (T_a_ = 72 °C). All plasmids were verified by Sanger sequencing.

### Cell culture and transfection

HEK293T cells were maintained in Dulbecco’s modified Eagle’s medium (DMEM) – high glucose (Sigma-Aldrich) supplemented with 10% fetal bovine serum (Sigma-Aldrich), 2 mM L-glutamine (Sigma-Aldrich), 100 U/ml penicillin and 100 µg/ml streptomycin (Sigma-Aldrich). For expression of WT and mutant G_s_α, 2.5-4.0 × 10^5^ HEK293T cells were seeded per well of a 6-well plate and transfected the following day with 3 µl GeneJuice Transfection Reagent (Sigma-Aldrich) and 1 µg plasmid DNA for 48 h according to the manufacturer’s instructions.

### cAMP assay

Cell lysate cAMP levels were compared using the cAMP-Glo Max Assay Kit (Promega) according to the manufacturer’s instructions. Briefly, 1 × 10^4^ HEK293T cells were seeded per well of a 96-well plate and incubated overnight. The following day, cells were washed with PBS and incubated in 40 µl complete induction buffer, consisting of 500 µM 3-isobutyl-1-methylxanthine (IBMX) (Sigma-Aldrich), 100 µΜ Ro 20-1724 (Sigma-Aldrich) and 25 mM MgCl_2_ in Opti-MEM (Gibco), with or without 10 µΜ forskolin (Bio-Techne) as indicated, for 15 min at 37 °C/5% CO_2_. 10 µl of cAMP Detection Solution was then added to each well, mixed by shaking for 1–2 min, and incubated at room temperature for 20 min. For development of luminescence signal, 50 µl of Kinase-Glo Reagent was added to each well, mixed by shaking for 1–2 min, and plates were incubated at room temperature for 10 min. Immediately following the final incubation, 100 µl sample from each well was transferred to a white 96-well microplate (Greiner Bio-One) and luminescence was measured using a CLARIOstar Plus instrument (BMG Labtech).

### Statistical analysis

Results are expressed as means with 95% confidence intervals and were analysed using the Kruskal-Wallis test, followed by pairwise Wilcoxon rank sum tests with Bonferroni correction for multiple tests. Differences were considered significant when p-value < 0.05.

## Electronic supplementary material

Below is the link to the electronic supplementary material.

Supplementary Material 1

Supplementary Material 2

Supplementary Material 3

Supplementary Material 4

Supplementary Material 5

Supplementary Material 6

Supplementary Material 7

Supplementary Material 8

Supplementary Material 9

## Data Availability

The RNA-seq data used in this study have been deposited in the NCBI Sequence Read Archive (SRA) https://www.ncbi.nlm.nih.gov/geo/ with reference number GSE276271. Individual files are numbered GSM8494432 to GSM8494446 inclusive. Other data that support the findings of this study are available from the corresponding author upon reasonable request.
