# Supplementary material for: Transcriptomic analysis reveals a critical role for activating Gsα mutations in spontaneous feline hyperthyroidism
Source: Sci Rep. 2024 Nov 20;14:28749. doi: 10.1038/s41598-024-79564-z (PMC11579033; doi:10.1038/s41598-024-79564-z)
Supplement: Supplementary file 1 — Supplementary Material 1 [file 41598_2024_79564_MOESM1_ESM.docx]

# Supplementary figure legends

## Figure S1

(**a**) Boxplots showing normalised expression of genes encoding thyroid peroxidase (*TPO*), sodium/iodide symporter (*SLC5A5*), dual oxidase 1 (*DUOX1*) and TSH receptor (*TSHR*). (**b**) Boxplots showing normalised expression of potential alternative transporters of iodide across the thyroid epithelial cell apical membrane. (**c**) Boxplot showing normalised expression of the gene encoding the thyroid hormone precursor thyroglobulin (*TG*). Individual samples are plotted by group (ET and HT) and coloured according to thyroid and treatment status. Expression is plotted as log_2_ transformed TMM-normalised counts per million mapped reads (CPM). Genes with significantly different expression between HT and ET samples are marked with * (adjusted p-value < 0.05), and genes with adjusted p-value > 0.05 are marked NS (not significant).

## Figure S2

(**a**) and (**b**) Low magnification scans of (**a**) ET04 (Grade 1, ‘Low’) and (**b**) HT06 (Grade 6, ‘High’) thyroid histopathology sections shown in Fig. 2a. (**c**) High magnification scans of thyroid histopathology sections from (*top*) ET04 (Grade 1, ‘Low’), (*middle*) HT01 (Grade 4, ‘Inter.’) and (*bottom*) HT06 (Grade 6, ‘High’). (**d**) and (**e**) Results of GO:BP enrichment analysis for DEG clusters from Fig. 2b with highest expression in (**d**) intermediate grade samples (including HT02 – adenocarcinoma and HT03 – ungraded) and (**e**) high grade samples.

## Figure S3

(**a**) Plot of log_2_ fold changes for DEGs in the full analysis (all HT vs ET samples) against their log_2_ fold changes in the focused analysis (high-grade HT vs ET). Each point represents a DEG, and points are coloured by their cluster membership in Fig. 2b. Inset, top left: overlap of each DEG cluster from Fig. 2b (all HT vs ET samples) with DEGs in the focused analysis (high-grade HT vs ET). (**b**) and (**c**) Results of GO:BP enrichment analysis for DEGs with (**b**) higher and (**c**) lower expression in high-grade HT samples compared with ET samples. (**d**) and (**e**) Gene set enrichment analysis (GSEA) plots showing enrichment of MSigDB Hallmark gene sets (**d**) oxidative phosphorylation and (**e**) epithelial-mesenchymal transition (EMT) in feline genes ranked by difference in normalised expression between high-grade HT and ET samples. FDR – false discovery rate, NES – normalised enrichment score. (**f**) Boxplots showing normalised expression of *VIM* (vimentin) and *CDH1* (E-cadherin), key markers of mesenchymal and epithelial cells, respectively. Individual samples are plotted according to sample group in Fig. 2 (L – ‘Low’, I – ‘Inter.’, H – ‘High’), and coloured by grade assigned during histopathological assessment.

## Figure S4

(**a**) STRING protein-protein interaction (PPI) network for 2,290 DEGs with absolute fold change > 2. Nodes are coloured by log_2_ fold change (high-grade HT vs ET) and node size is proportional to the node degree (number of connected edges). (**b**) Node degree versus BetweennessCentrality for the PPI network in (**a**). Each point represents a node (i.e. DEG) and is coloured by log_2_ fold change in the high-grade HT vs ET analysis. The top 10 nodes, ranked by node degree, are labelled. (**c**), (**d**) and (**e**) Clusters of highly connected nodes identified using the Markov Cluster (MCL) algorithm. Each cluster is labelled by the most strongly enriched GO:BP gene set for nodes in that cluster. (**c**) Mitochondrial respiration and fatty acid metabolism, (**d**) multiple signalling pathways involved in tissue patterning, morphogenesis and development, and (**e**) inflammatory cytokine and interferon signalling.

## Figure S5

(**a**) Genotypes of *TSHR* variants detected in RNA-seq reads from HT and ET samples. Genomic coordinates are for the felCat9 reference genome (see Methods). Variants are labelled with the resulting amino acid substitution in TSHR, and with the variant ID from Watson et al.^1^ where applicable. (**b**) Multiple sequence alignment of protein sequences for feline, human and bovine G_s_α. Regions highlighted orange are identical between all three species. The transmembrane domain is bounded by the black box, and red boxes mark positions of the ‘switch’ regions involved in GTP/GDP exchange^2^. Mutation of residues highlighted blue has previously been reported to activate G_s_α, and mutation of the residue highlighted magenta is novel to this study.


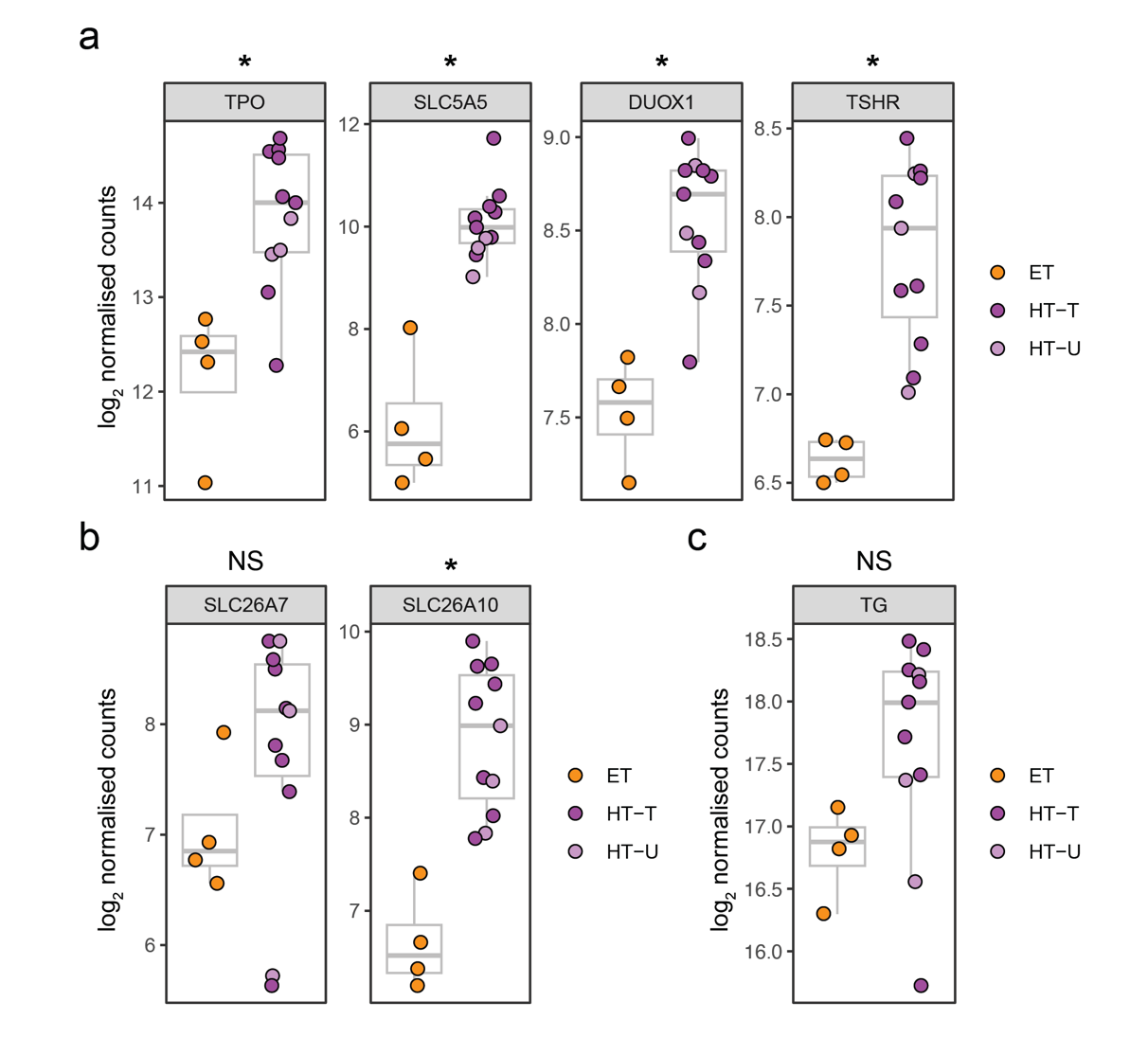


**Figure S1**


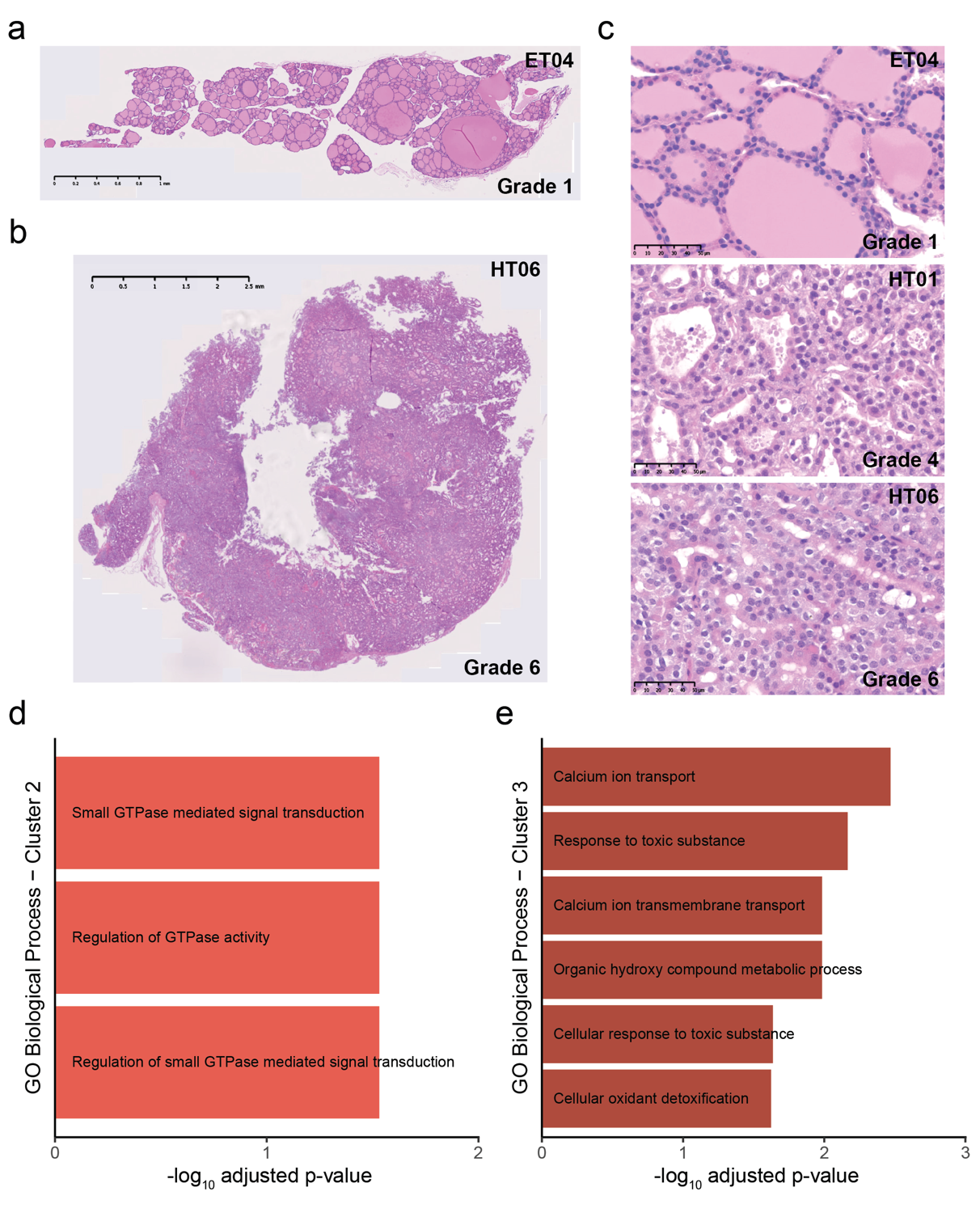


**Figure S2**


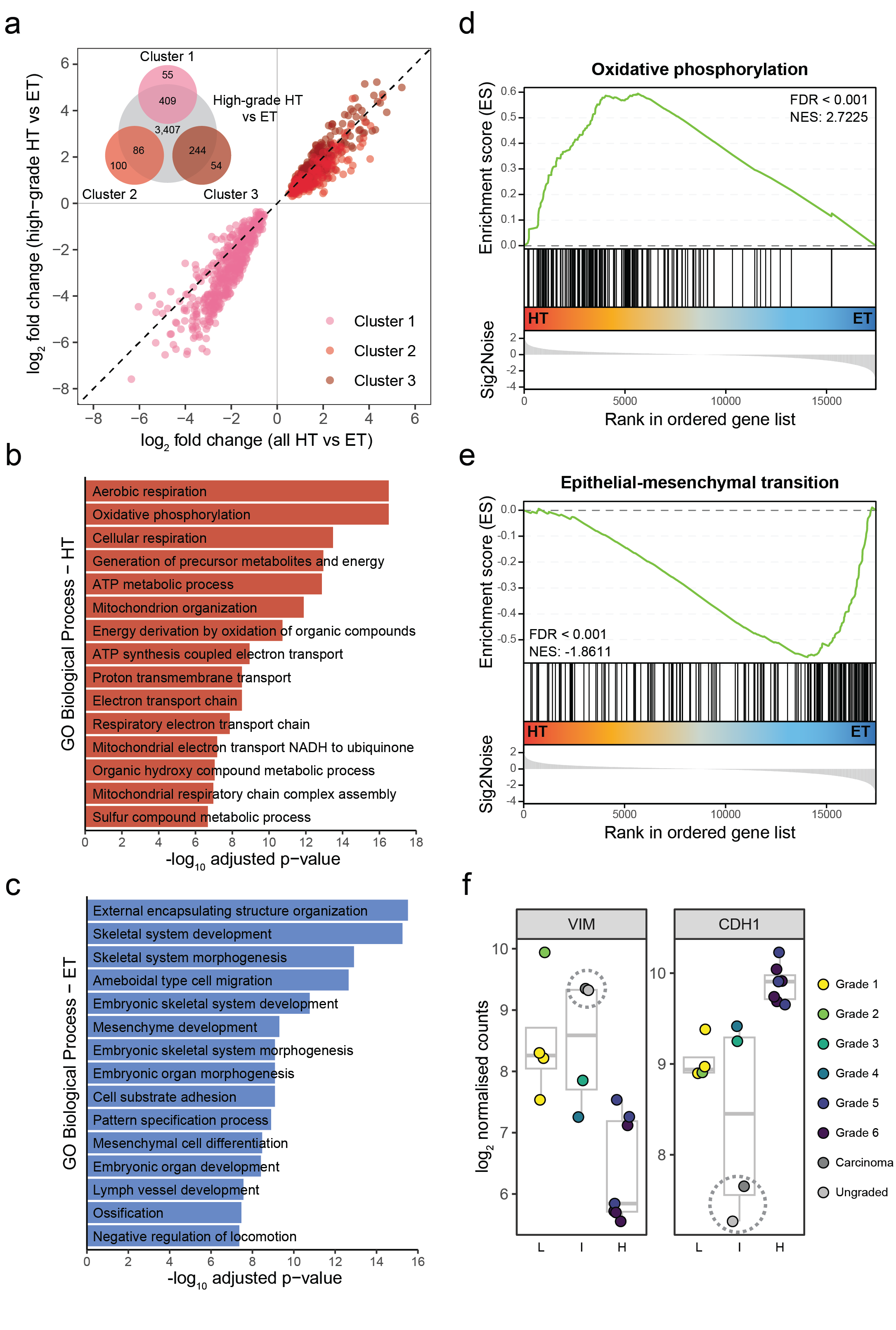


**Figure S3**


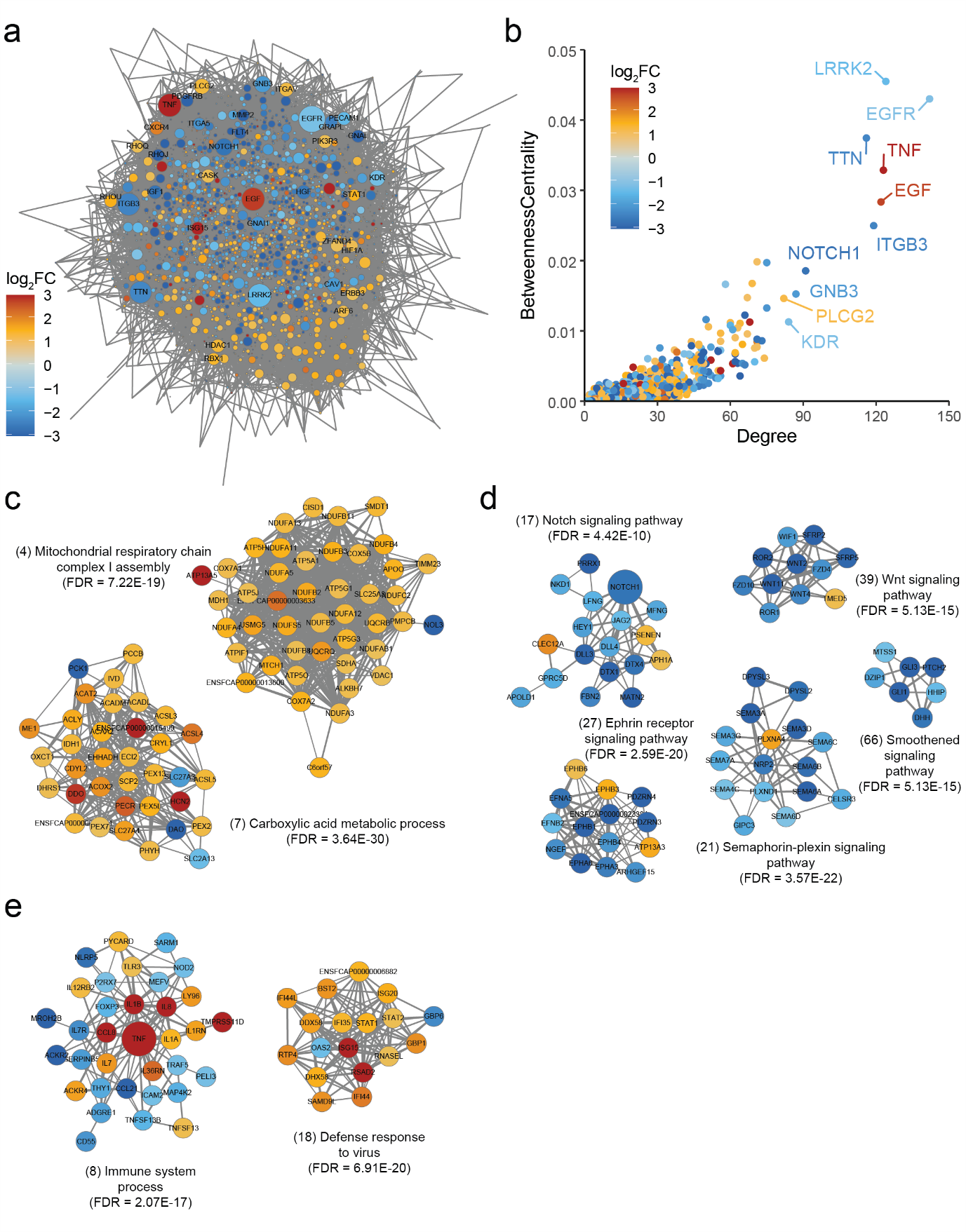


**Figure S4**

_
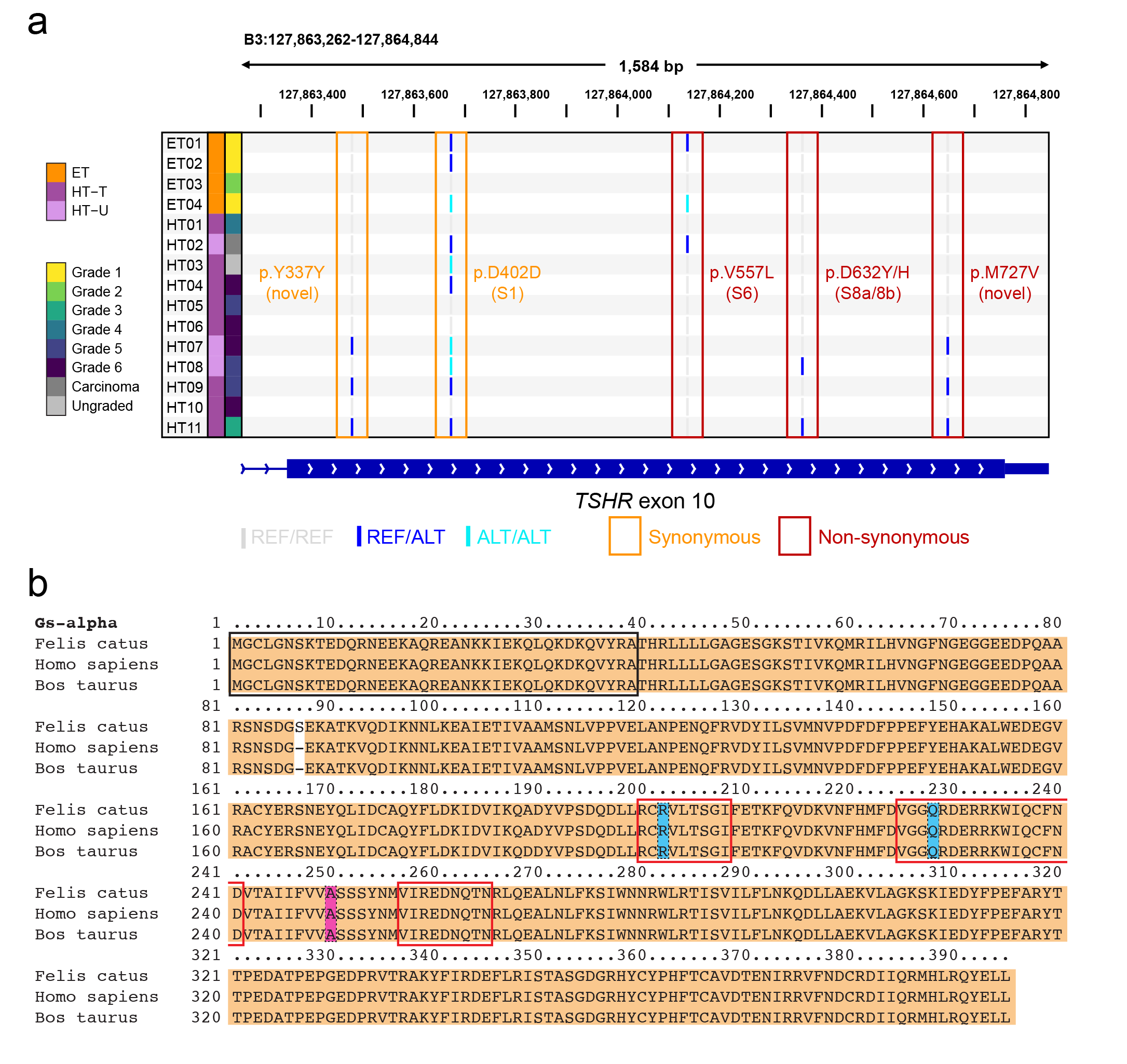
_

**Figure S5**
